# Interleukin33 deficiency causes tau abnormality and neurodegeneration with Alzheimer-like symptoms in aged mice

**DOI:** 10.1038/tp.2017.142

**Published:** 2017-07-04

**Authors:** C Carlock, J Wu, J Shim, I Moreno-Gonzalez, M R Pitcher, J Hicks, A Suzuki, J Iwata, J Quevado, Y Lou

**Affiliations:** 1 Department of Diagnostic Sciences, School of Dentistry, McGovern School of Medicine, University of Texas Health Science Center at Houston, Houston, TX, USA; 2 Department of Neurology, McGovern School of Medicine, University of Texas Health Science Center at Houston, Houston, TX, USA; 3 Department of Psychiatry and Behavioral Sciences, McGovern School of Medicine, University of Texas Health Science Center at Houston, Houston, TX, USA; 4 Department of Pathology, Baylor College of Medicine, Houston, TX, USA; 5 Laboratory of Neurosciences, Graduate Program in Health Sciences, Health Sciences Unit, University of Southern Santa Catarina, Criciúma, Brazil

## Abstract

Late-onset Alzheimer’s disease (AD) remains a medical mystery. Recent studies have linked it to impaired repair of aged neurons. Potential involvement of interleukin33 (IL33) in AD has been reported. Here we show that IL33, which was expressed by up to 75% astrocytes in the aged brains, was critical for repair of aged neurons. Mice lacking *Il33* gene (*Il33*^*−/−*^) developed AD-like disease after 60–80 weeks, which was characterized by tau abnormality and a heavy loss of neurons/neurites in the cerebral cortex and hippocampus accompanied with cognition/memory impairment. We detected an abrupt aging surge in the cortical and hippocampal neurons at middle age (40 weeks). To counter the aging surge, wild-type mice rapidly upregulated repair of DNA double-strand breaks (DSBs) and autophagic clearance of cellular wastes in these neurons. *Il33*^*−/−*^ mice failed to do so, but instead went on to develop rapid accumulation of abnormal tau, massive DSBs and abnormal autophagic vacuoles in these neurons. Thus, uncontrolled neuronal aging surge at middle age due to lack of IL33 resulted in neurodegeneration and late-onset AD-like symptome in *Il33*^*−/−*^ mice. Our study also suggests that the aging surge is a time to search for biomarkers for early diagnosis of AD before massive neuron loss.

## Introduction

Late-onset Alzheimer’s disease (AD), which is an increasing socioeconomic burden worldwide, remains a medical mystery.^1^ However, recent studies have linked this disease to the impairment in rejuvenation or repair of aged neurons.^2, 3, 4, 5, 6^ These mechanisms include DNA damage repairing and autophagic elimination of metabolic wastes.^7, 8, 9^ Neuron’s aberrant reentry into cell cycle in AD may de-regulate these mechanisms, resulting in neuron death.^10^ Deficiencies in cytokines or other immune molecules have also reportedly implicated in these rejuvenation mechanisms and neurodegenerative diseases including AD.^11, 12, 13^ Expression of various cytokines such as IL-1β and TNFα in AD patients suggests their roles in AD pathogenesis.^13, 14^ However, it remains to be determined if these cytokines may act as protective or inflammatory roles.

Interleukin33 (IL33), which is often detected as a nuclear protein, is a member of the interleukin1 cytokine family. It acts as mature cytokine after cleavage with ST2 as its receptor. Beyond its multifunction in immune defense, IL33 also plays a role in the injury healing in central nervous system and other diseases.^15, 16, 17, 18, 19^ IL33 has been genetically linked to human AD.^20^ Injection of recombinant IL33 shows a beneficial effect in mouse AD models.^21^ Constitutive expression of IL33 in a wide range of tissues including the brain suggests its potential roles beyond immune defense.^22, 23, 24, 25, 26, 27^ Our previous study has demonstrated one such role for IL33 in tissue homeostasis in degenerative ovarian tissue.^28, 29^ In the present study, we investigated role of IL33 in tissue homeostasis in the brain. We found that IL33 was critical for repair of aged neurons. Its deficiency caused tau abnormality and late-onset of neurodegeneration in the cerebral cortex and hippocampus, accompanied with AD-like cognition and memory impairment.

## Materials and methods

### Mice and their treatment

C57BL/6 (B6) mice were purchased from Harlan (Indianapolis, IN, USA). *Il33*^tm1(KOMP)Vlcg^ (*Il33*^*−/−*^) mouse strain was created (WWW.KOMP.org) and characterized.^29, 30^ The *Il33*^*−/−*^ strain shows generally normal without any developmental defects.^29, 31^ All animal procedures in this study were approved by institutional animal welfare committee. Mice were randomly selected for all experiments, and were tested with the group allocation blind to investigators; data were assembled after testing for each group for statistical analysis. Mice were perfused with room temperature PBS followed 2% paraformaldehyde before brains were harvested. In some cases, fresh brains were used.

### Histology and electron microscopy

Brain tissues, fixed through perfusion, were embedded in paraffin and used for routine histology, including hematoxylin–eosin (H–E) staining, Bielschowsky silver staining and crystal violet staining. Three samples per group were processed for transmission electron microscopy following an established method.^29^

### Behavioral tests

Four behavioral tests were performed for assessment of cognition/memory impairment associated with AD in mice.^32, 33, 34^ Locomotor activities (horizontal and vertical) test was performed in a computer-controlled activity cage (Ugo Basile, Monvalle, Italy) for an untrained mouse in the open testing chamber (54 × 50 × 37 cm) for recording its horizontal and vertical motions for 30 min. For habituation test, a mouse was placed in an open-field (60 × 40 cm) surrounded by 50 cm high plexi-glass wall, and allowed to explore freely for 5 min at day 1 and day 2 with numbers of its movements, that is crossing and rearing, recorded. Fear-based passive avoidance test was performed in Shocker-with-Scrambler behavioral chamber (PanLab, Barcelona, Spain). Mice received an electric shock (0.8 mA × 2 s) as training. The trained mice returned to the white chamber at day 1 and day 7 with their latency time for entering dark chamber (limited to 3 min) recorded. Rotarod performance test was carried out on an automatic instrument (Rotamex 4, Columbus Instrument, Columbus, OH, USA).

### Bromodeoxyuridine incorporation test

Bromodeoxyuridine (BrdU; BD Biosciences, Franklin Lakes, NJ, USA) was dissolved in sterile DPBS at 10 mg ml^−1^. Each mouse received intra-peritoneal injection of BrdU solution at a dose of 1.5 mg per 25 g bodyweight in 150 μl of solution. The mice were killed 24 h late and perfused, and their brains or other tissues removed for detection of incorporated BrdU by immunofluorescence using an anti-BrdU antibody.

### Cerebral cortical homogenate, and fractionation of nuclei and organelles by gradient centrifugation

Fresh cortexes were homogenized on ice in an extraction buffer containing a protease inhibitor cocktail (Sigma-Aldrich, St Louis, MO, USA). After centrifugation at 5000 *g* for 15 min at 4 °C, the supernatant was removed, and protein concentration measured. For nuclear fractionation, a published method with modifications was followed.^35^ Whole cortex was cut into 2 × 2 mm^3^ in cold PBS, and gently homogenized in a glass homogenizer by B pestle (Wheaton Dounce tissue grinder, Millville, NJ, USA). Crude nuclear fraction was recovered by centrifugation, laid on the top of five-layer sucrose gradient (2.0, 2.2, 2.4, 2.6 and 2.8 M), and centrifugation in a swing basket rotor at 53 500 *g* for 45 min. Each nuclear fraction was recovered from the interfaces: astrocyte nuclei on interface of 2.4 and 2.6 M, and neuronal nuclei 2.6 and 2.8 M. A small portion was used for microscopy to confirm expected nuclear morphology for each fraction. A published method was followed for fractionation of autophagosomes, autolysosomes and lysosomes of cortical tissues.^36, 37^ Cortical homogenate was centrifuged at 6500 *g* for 5 min, and the supernatant was further centrifuged at 17 000 *g* for 10 min to pellet the subcellular compartments, followed by Nycodenz (Sigma-Aldrich) discontinuous gradient (10, 20, 24, 26, 50%) at 25 000 r.p.m. for 4 h. Fractions recovered from interfaces of 10–20% and 20–24%, contained autophagosomes and autolysosomes, respectively. All procedures were carried out at 4 °C.

### Antibodies

Following antibodies were used in this study: biotin goat anti-mouse IL33, rat anti-mouse IL33, biotin rabbit anti-LC3, Alex Fluor555 rabbit anti-LC3, rabbit anti-GFAP, mouse anti-tubulin β3, rabbit anti-BrdU, rabbit anti-PγH2AX, rabbit anti-ubiquitin, rabbit anti-amyloid β antibody, mouse anti-phosphor-tau AT8, PHF1 and MC1, FITC-labeled anti-α-actin. Secondary reagents included Alexa-555, Alexa-594 and Alexa-647-labeled (Life Technologies, Carlsbad, CA, USA) and PE-labeled streptavidin. Biotin/avidin and anti-mouse CD16/32 were used for blocking non-specific IgG binding. Immunoglobulin isotypes were used as negative controls (BD Biosciences).

### Western blot

Proteins were quantitated (Epoch Gen5, BioTek, Winooski, VT, USA), and mixed at 1:1 with SDS sample buffer. Ten micrograms of protein were loaded on a SDS-polyacrylamide gel electrophoresis of various concentrations depending on size of target protein, and ran at a constant current. After transfer, the membrane (Immobilon-P PVDF, Millipore, Billerica, MA, USA) was used for immunostaining. Anti-α-actin mouse monoclonal antibody (AC-15, Sigma) was simultaneously added with the antibody to the target protein. The membrane was further incubated with IRDye 800CW-labeled secondary antibody for target protein and IRDye 680LT anti-mouse IgG antibody (LI-COR, Lincoln, NE, USA). The membrane was simultaneously scanned at both wave lengths on an infrared fluorescence scanner (Odyssey, LI-COR), with target protein as green and control α-actin as red.

### Immunofluorescence, terminal-deoxynucleotidyl-transferase dUTP nick-end labeling, immunohistochemistry and quantitation of cells on sections

Frozen sections were cut from brain tissues, and were blocked in 3% BSA with CD16/32 antibodies. If biotin-labeled antibodies were to be used, a biotin and avidin blocking step was added (Vector BioLab, Philadelphia, PA, USA). Up to four colors, that is, green (FITC or Alex488), red (TRITC, PE, Alex594/555), false purple (APC or Alex647) and blue (DAPI) were applied for each sections. In some cases, 20 μm sections were used for 3D scan through Z-stack in confocal microscopy. For terminal-deoxynucleotidyl-transferase dUTP nick-end labeling (TUNEL) staining, a kit (*In Situ* Cell Death Detection Kit, Fluorescein, Roche, Nutley, NJ, USA) was used. The tissue sections observed by a confocal microscope (Nikon, Tokyo, Japan, C2^+^ Eclipse T*i*). In some cases, whole-brain sections were automatically scanned and merged (Nikon Eclipes N*i*). Digital images were analyzed with NIS Elements 3.2 (Nikon) for fluorescent intensity as integrated optical density, cellular area or cell numbers. For immunohistochemistry, secondary reagents (avidin-peroxidase or peroxidase-conjugated secondary antibody) were used to generate a brown deposition using DAB as a substrate in the presence of H_2_O_2_.

### Statistics

Paired (habituation tests) or unpaired *T*-tests (others) were used for comparison between two groups. For three groups, one-way analysis of variance was performed. Before pooling data from multiple individuals, data from each were statistically compared to rule out any differences among them. Linear regression test was used for analysis of correlations between ages and TUNEL densities, between age and locomotor activities, in wild-type (WT) and *Il33*^*−/−*^ mice, respectively; *r*^*2*^ and *P*-value for deviation from zero was calculated for each progression. Finally, slopes of the two linear progressions were compared for statistical significance. In addition, time courses for TUNEL densities in *Il33*^*−/−*^ or WT mice were constructed by non-linear four-parameter progression. Statistical significances were indicated by *(*P*<0.05), **(*P*<0.01) or ***(*P*<0.001).

## Results

### IL33 expression in astrocytes increases with age

**Figure 1:**
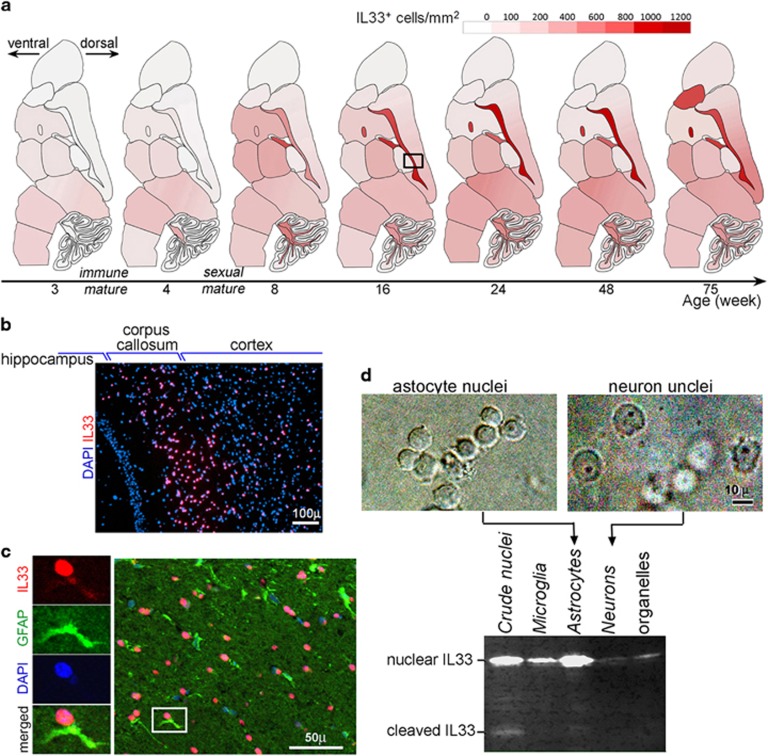
Interleukin33 (IL33) expression in astrocytes in brains increases with age. (**a**) Distribution pattern of nuclear IL33^+^ cells in the brains at different ages. Densities were calculated from two to three mice for each age. Scale for density of IL33^+^ cells per mm^2^ is shown. (**b**) Immunofluorescence of nuclear IL33^+^ cells (red) in the boxed area in **a**. (**c**) Immunofluorescence reveals nuclear IL33 (red) in astrocytes identified by GFAP (green). Left panels show each fluorescent channel of the boxed cell at right panel. (**d**) Western blot detects (lower panel) abundant IL33 protein in astrocyte nuclei. Both nuclear IL33 and cleaved cytokine IL33 were detected. Phase-contrast images for fractionated nuclei are shown on the top.

Expression of IL33 in brains has been well studied.^23, 24, 25, 26^ We especially examined spatial and temporal expression pattern of IL33 in mouse brains from 3 to 75 weeks. Nuclear IL33 expression increased with age well past both immune and sexual maturity (Figure 1a). The highest density of nuclear IL33^+^ cells (>1000 cells per mm^2^ or up to 75% of cells) was in the regions rich in nervous fibers of aged mice (Figure 1b). Immunofluorescence revealed that IL33 was often detected in nuclei of astrocytes, which were identified by GFAP (Figure 1c). Western blots of the fractionated brain cell nuclei confirmed that astrocyte nuclei were the most abundant in IL33 (Figure 1d). Thus, IL33 was primarily expressed by astrocytes, which is in agreement with several previous studies.^15, 26^ A cleaved IL33 (19 kDa) was also detectable (Figure 1d), suggesting a release of cytokine IL33 in normal brains. Increasing extensive expression of IL33 with age and release of cytokine IL33 in normal brains are un-proportional to central nervous system immune privilege, suggesting IL33’s role in central nervous system tissue homeostasis during aging.

### Il33^−/−^ mice develop tau abnormality and late-onset neurodegeneration in cerebral cortex and hippocampus

**Figure 2:**
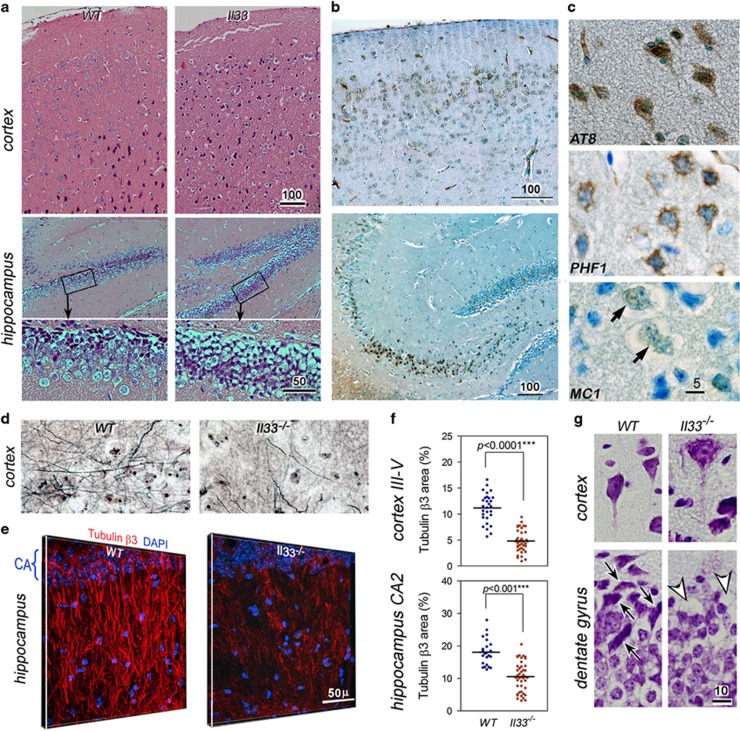
*Il33*^*−/−*^ mice develop late-onset neurodegeneration and abnormal tau accumulation in the cerebral cortex and hippocampus. (**a**) Cortex and hippocampus from a representative 60-week *Il33*^*−/−*^ mouse display neurodegeneration as compared to a wild-type (WT) mouse; enlarged boxed areas are shown below. Total seven mice for each group were examined with similar results. (**b**) Immunohistochemistry reveals heavy accumulation of paired helical fragment (PHF1) tau in cortical and hippocampal neurons in a representative 70-week *Il33*^*−/−*^ mouse (*n*=7). (**c**) Immunohistochemistry reveals cellular abnormal tau (AT8, PHF and insoluble tau MC1) in neurons. Arrows indicate neuron with MC1. (**d**) Silver stain of 30-μm section shows greatly reduced neurite networks in the cortex of a representative 70 weeks of *Il33*^*−/−*^ mouse (*n*=5) as compared to a WT littermate of the same age (*n*=6). (**e**) Three-dimensional immunofluorescence on protein tubulin β3 reveals loss of neurite tufts of hippocampus in a 65-week *Il33*^*−/−*^mouse (*n*=5) as compared to an age-matched WT mouse (*n*=5). (**f**) Statistical summary of tubulin β3 area in WT and *Il33*^*−/−*^ brains. (**g**) Crystal violet staining shows vacuoles in soma and axon of pyramid neurons (upper right), or empty spaces (arrow heads, lower right) in dentate gyrus of an *Il33*^*−/−*^mouse (*n*=3). Note many neurons (arrows in lower left panel) are present in the same locations in a WT mouse. Bar unit=μm.

**Figure 3:**
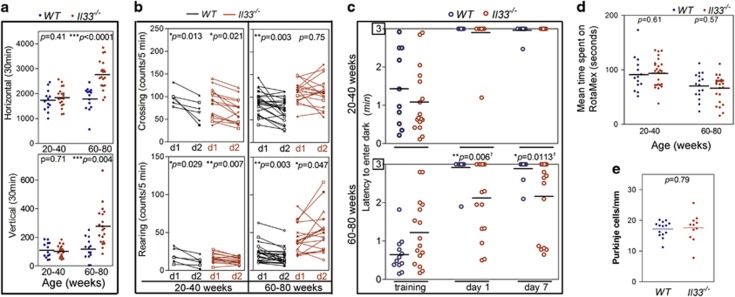
*Il33*^*−/−*^ mice develop cognition/memory impairments after 60–80 weeks. (**a**) Locomotor activity (vertical and horizontal) tests reveal elevated activities in old *Il33*^*−/−*^ mice (60–80 weeks). (**b**) Habituation test reveals no decline in exploring activities (both rearing and crossing) at day 2 (d2) in aged *Il33*^*−/−*^ mice (60–80 weeks) after training at day 1 (d1). Wild-type (WT) or young *Il33*^*−/−*^ mice displayed significantly lower exploration activities at day 2. Activities were shown for each individual. (**c**) Electric shock-based passive avoidance test shows more frequent re-entry into dark chamber among aged *Il33*^*−/−*^ mice (60–80 weeks). Data are shown for each individual. (**d**) Rotamex test show no differences between either young or old WT and *Il33*^*−/−*^ mice. Data are shown for each individual. (**e**) Old *Il33*^*−/−*^ mice show a similar density of Purkinje cells in cerebella as compared to age-matched WT mice; *n*=5. Comparisons were made by two-tailed *t*-test (**a**,**d**,**e**), paired *t*-test (**b**) and Welch’s *t*-test (**c**).

A mouse strain with *Il33* gene deleted (*Il33*^*−/−*^) has been generated and characterized.^29^ We compared their brains to WT littermates. *Il33*^*−/−*^ brain were normal before 40 weeks, but showed significant neurodegeneration in the cerebral cortex and hippocampus after 60 weeks (Figure 2a). Heavy deposition of abnormal tau, that is, hyper-phosphorylated, paired helical fragment (PHF), and insoluble tau was detected in cortical and hippocampal neurons in six out of seven mice of 65–80 weeks (Figures 2b and c). Both silver staining on 30-μm sections and immunofluorescence on tubulin β3 revealed a heavy loss of neurites/neurons. The loss is well exemplified by disappearance of both normal cortical layers and hippocampal apical dendrite tufts constituted by tubulin β3 in *Il33*^*−/−*^ mice (Figures 2d and e, Supplementary Figures 1a and b). Quantitation showed that tubulin β3 density in *Il33*^*−/−*^ mice reduced to 55% and 37% of the WT mice in the cortex and hippocampus, respectively (Figure 1f). Vacuoles were often observed in neuronal soma and neurites as early as 40 weeks (Figure 2g). Often open oval-shaped empty space was left after the loss of neurons (Figure 2g). These changes prompted us to test for any behavioral changes in *Il33*^*−/−*^ mice. Increased locomotor activities are associated with murine AD.^38, 39^ *Il33*^*−/−*^ mice showed an age-related increase in locomotor activities especially after 60 weeks (Figure 3a, Supplementary Figure 1c). In habituation tests, old *Il33*^*−/−*^ mice (60–80 weeks) did not display a decline in exploration activities post training as age-matched WT mice (Figure 3b). Fear-based passive avoidance test is often used to assess behavioral changes associated with AD or neurodegenerative diseases.^40^ This test revealed a significantly higher re-entry rate into the dark chamber post electric shock training in old *Il33*^*−/−*^ mice, suggesting loss of short memory (Figure 3c). However, *Il33*^*−/−*^ mice under 40 weeks did not show any behavioral changes as compared with WT mice (Figures 3a–c). Thus, *Il33*^*−/−*^ mice began to develop cognition/memory impairments after 60–80 weeks. Interestingly, old *Il33*^*−/−*^ mice showed no differences from age-matched WT mice in either motor function assessment or Purkinje cell density, suggesting that their cerebella were relatively unaffected (Figures 3d and e, Supplementary Figure 1d).

### Il33^*−/−*^ mice fail to repair stressed neurons after an abrupt aging surge at middle age

**Figure 4:**
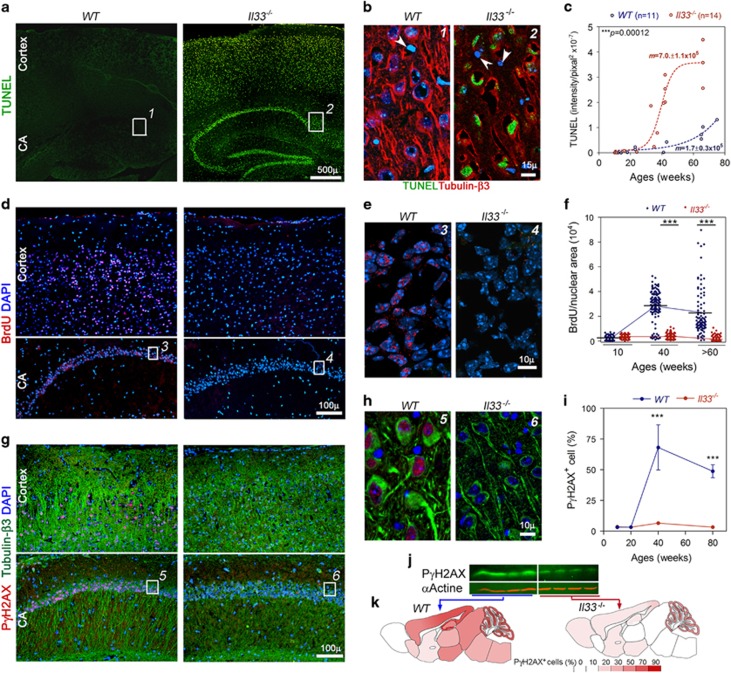
*Il33*^*−/−*^ mice fail to initiate the repair of rapidly increasing DNA double-strand breaks (DSBs) in the cortical and hippocampal neurons after 40 weeks. (**a**) Terminal-deoxynucleotidyl-transferase dUTP nick-end labeling (TUNEL) reveals the accumulation of DSBs (green) in neurons in the cortex and hippocampus (CA) of a 40-week *Il33*^*−/−*^ mouse, in comparison to a 40-week wild-type (WT) mouse. (**b**) Co-staining of TUNEL (green) with tubulin β3 (red) for numbered boxes in **a** shows that neurons are TUNEL^+^, but glial cells (arrowheads) are TUNEL negative. (**c**) Time course of TUNEL density in brains reveals a rapid increase in TUNEL^+^ neurons in *Il33*^*−/−*^ mice between 35 and 40 weeks. TUNEL^+^ density in each brain is quantitated as total fluorescent intensity/brain section area. Liner progression was used for comparison between WT and *Il33*^*−/−*^ group, and four-parameter progression for construction of curves. (**d**) Immunofluorescence demonstrates incorporated BrdU (red) in the nuclei of WT (left) but not in *Il33*^*−/−*^ (right) neurons in cortex and hippocampus at 40 weeks (*n*=4). (**e**) Enlarged numbered boxed areas in **d** showed nuclear BrdU (red) only in WT neuronal nuclei. (**f**) Quantitation of BrdU incorporation in hippocampal dentate gyrus (*n*=4 per age). Each dot represents BrdU incorporation in one neuron (fluorescent intensity/nuclear area). (**g**) Immunofluorescence reveals PγH2AX (red) in the cortex and hippocampus (CA) of WT mice, but not in *Il33*^*−/−*^ mice, at 40 weeks (*n*=4). Note heavy loss of neurons/neurites in *Il33*^*−/−*^ mice as revealed by co-staining for tubulin β3 (green). (**h**) Enlarged-numbered-boxed areas in **g** show PγH2AX (red) only in WT neuronal nuclei (green). (**i**) Summary of PγH2AX^+^ neuron density (%) in dentate gyrus at various ages (*n*=3 per group). (**j**) Western blot on cortical proteins shows reduced PγH2AX in *Il33*^*−/−*^ mice at 40 weeks. (**k**) Distribution of PγH2AX^+^ cells in the mouse brains at 40 weeks (*n*=3). Cell densities (%) are indicated by color scale.

We next explored what had led to neurodegeneration in the cortex and hippocampus in old *Il33*^*−/−*^ mice. We first detected an overwhelmingly large number of TUNEL^+^ nuclei in the cortex and hippocampus in aged *Il33*^*−/−*^ mice (Figure 4a). Notably, ~90% of the cells in the dentate gyrus and CA region of hippocampus were TUNEL^+^. Co-staining with tubulin β3 revealed that the TUNEL^+^ cells were neurons (Figure 4b). Brain TUNEL^+^ neurons have been detected in human AD autopsy and animal models for neurodegenerative diseases.^41, 42^ However, the nature of these TUNEL^+^ neurons remains ambiguous. We compared these TUNEL^+^ neurons with apoptotic ovarian cells during atresia of the same individuals.^29^ TUNEL intensity in neurons was only 1/20 to 1/40 of that of those apoptotic cells without any detectable caspases or DNA condensation (Supplementary Figures 2a and b). TUNEL can also detect genomic DNA double-strand breaks (DSBs). Thus, TUNEL^+^ in neuronal nuclei of *Il33*^*−/−*^ mice indicated accumulation of a large number of DSBs. Whole-brain sections were scanned for calculating TUNEL density to quantitate DSBs. A rapid increase in DSBs was observed in a 35–40-week window in *Il33*^*−/−*^ brains (Figure 4c). The DSBs continued to increase with age thereafter, but at a slower rate. In contrast, only a few TUNEL^+^ cells were sporadically present in WT mice even after 60 weeks.

Oxidative stress may induce chronic neuronal death in AD.^10, 43, 44^ It causes apurinic/apyrimidinic (AP) site DNA lesion, leading to DSBs. However, AP lesion sites in *Il33*^*−/−*^ cortex at 40 weeks was comparable to WT mice (Supplementary Figure 2c). We next asked whether increased DSBs were due to a failure in DNA repair in *Il33*^*−/−*^ mice. BrdU incorporation is a measurement for DSB repairing. After injected with BrdU, young WT mice (up to 20 weeks) showed no BrdU incorporation in their brain. However, animals over 40 weeks showed numerous neurons with nuclear BrdU in the cortex and hippocampus (Figures 4d–f), where TUNEL positive cells were extensive in *Il33*^*−/−*^ mice. In contrast, BrdU incorporation was not observed at any ages in *Il33*^*−/−*^ mice, suggesting a failure in DSB repairing in *Il33*^*−/−*^neurons (Figures 4d–f). When a DSB occurs, histone γH2AX will be phosphorylated for recruiting repair machinery. Thus, phosphorylated γH2AX (PγH2AX) is a marker for both DSBs and the initiation of repair.^45^ Similar to BrdU incorporation distribution pattern, immunofluorescence detected PγHA2X in the nuclei of 80% of neurons in the cortex and 60% in the hippocampus of WT mice over 40 weeks (Figures 4g–i, Supplementary Figure 2d). PγH2AX was limited to neuronal nuclei (Figure 4h). In contrast, *Il33*^*−/−*^ mice of any ages showed nearly no PγH2AX^+^ neurons (Figure 4g–i, Supplementary Figure 2d). Western blot of cortical proteins also showed a significantly lower level of PγH2AX in *Il33*^*−/−*^ mice (Figure 4j). The distribution pattern of PγH2AX^+^neurons in the brain was very similar to that for BrdU in WT mice. The cortex and hippocampus of *Il33*^*−/−*^mice showed a substantial reduction in PγH2AX^+^ neuron density. Nearly 90% of neurons in the WT dentate gyrus were PγH2AX^+^, but close to zero in *Il33*^*−/−*^ mice (Figure 4k). Co-incidental appearance of DSBs as TUNEL^+^ in *Il33*^*−/−*^ mice with a rapid increase in DSB repairing in WT mice in the same cortex and hippocampus region at or after 40 weeks reveals a surge of aging process in these neurons, which accelerated DSB generation. A failure in repairing these DSBs after the aging surge at 40 weeks had led to DBS accumulation in neurons as TUNEL^+^ in *Il33*^*−/−*^ mice. Unrepaired DSBs are cytotoxic and have been implicated in neuronal death in human AD.^7, 8, 46, 47, 48^

**Figure 5:**
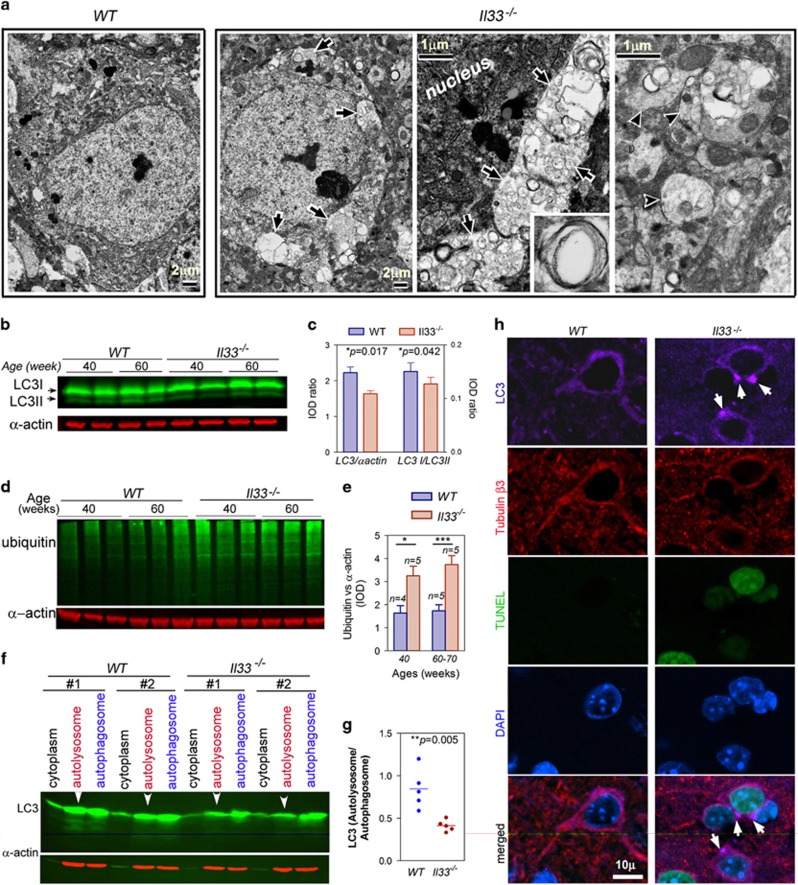
*Il33*^*−/−*^ mice fail to complete autophagic digestion in the neurons. (**a**) Electron microscopy reveals autophagic vesicles or vacuoles (arrows) in *Il33*^*−/−*^ cortical neurons. Inset shows a double-membraned autophagic vesicle. Vacuoles are also present in neurites (arrowheads in right most panel). Three mice per group were observed with similar results. (**b**) Western blots detect reduced LC3 in cortical proteins. Each lane is for one individual. (**c**) Quantified IOD of LC3 I, LC3 II and α-actin bands in cortical proteins at 40 weeks. Ratios between total LC3 vs α-actin (left) and LC3 II vs LC3 I (right) are shown; *n*=5. (**d**) Western blots reveal increased ubiquitinated proteins in the cortex of *Il33*^*−/−*^ mice. Each lane is for one individual. (**e**) Quantified total IOD for ubiquitinated proteins and α-actin. Ratios between IODs of ubiquitinated proteins and α-actin of cortical proteins are shown for wild-type (WT) and *Il33*^*−/−*^ mice at 40 weeks (*n*=5) or 60–70 weeks (*n*=5). (**f**) Western blot reveals a lower level of LC3 in autolysosomal fraction (arrowheads) in *Il33*^*−/−*^ mice as compared to WT mice. Note that LC3 levels in other fractions were comparable to WT mice. Two representative sets of samples are shown for each group. Autolysosomes and autophagosomes were isolated from the cortex. (**g**) Ratio of IODs between autolysosomal and autophagosomal LC3 in WT and *Il33*^*−/−*^ mice at 40 weeks. (**h**) Immunofluorescence shows LC3 aggregates (arrows in purple channel) in *Il33*^*−/−*^ neurons, which are distinguishable from WT neurons by TUNEL^+^ (green) nuclei and diminished tubulin β3 (red); *n*=3. IOD, integrated optical density.

Abnormal autophagy has been implicated in vacuolar neurodegenerations in central nervous system including AD.^2, 3, 49^ Autophagy deficiency has been linked to tau deposition and amyloid plaque.^50, 51^ We studied nature of neuronal vacuoles (Figure 2e). Electron microscopy first showed accumulation of numerous vesicles or vacuoles in both neural soma and neurites of *Il33*^*−/−*^ mice at 60 weeks with many of them double-membraned (Figure 5a, Supplementary Figure 2e). This indicates an abnormal accumulation of autophagosomes. LC3 and ubiquitinated proteins are often used as a measure for autophagy activities.^52^ LC3, a critical protein for the formation of autophagosomes, was reduced at 40 weeks prior to the onset of neuron loss in *Il33*^*−/−*^ mice (Figures 5b and c). Decrease in autophagy was also evidenced by an increase in ubiquitinated proteins in *Il33*^*−/−*^ mice (Figures 5d and e). However, reduced autophagic activities in *Il33*^*−/−*^ mice could not explain the accumulation of autophagosomes in *Il33*^*−/−*^ neurons. Cortical cells were fractionated into various organelles. A significantly lower quantity of autolysosomal LC3 was found in *Il33*^*−/−*^ mice (Figures 5f and g). This suggests a failure in fusion between autophagosomes and lysosomes in *Il33*^*−/−*^ neurons, leading to accumulation/aggregation of autophagosomes. Aggregated autophagosomes were detectable by immunofluorescence on LC3 in the neurons of *Il33*^*−/−*^ mice after 40 weeks (Figure 5h). Thus, *Il33*^*−/−*^ neurons also failed to complete autophagic digestion.

## Discussion

Current models for AD are largely transgenic animals, which overexpress mutant human amyloid precursor protein (APP), tau, or presenilin 1.^53, 54^ Those models have shed light on the role of aggregation of tau or amyloid β2 in interference with essential cellular mechanisms. However, amyloid plaques and tau deposition in late-onset AD are not associated with mutations. Thus, cause of late-onset AD remains unclear. Mounting evidence suggests a critical role of abnormal neuronal aging in late-onset AD.^2, 3, 4, 5^ *Il33*^*−/−*^ mice developed AD-like disease at old age due to impaired repair of aged neurons. Furthermore, the disease in our model resembles many pathological features of human late-onset AD. These shared features include late-onset neurodegeneration, heavy neuron loss in the cerebral cortex and hippocampus, tau abnormality and impaired cognition/memory at old age. Tau deposition is one of the most important hallmarks for human late-onset AD. To our knowledge, our model probably is the first one to show tau abnormality, which is un-related to mutant tau genes. Although amyloid plaques were not present in *Il33*^*−/−*^ mice, it is expected because murine APP lacks cleavage sties and hydrophobic residues for generating amyloid plaque.^54^

Neuronal aging process is also a result of accumulation of damaged molecules e.g. DSBs, reactive oxygen species and old organelles.^47^ Failed repair of DNA damage in aged neurons has been implicated in human AD.^7, 8, 9^ Our study revealed an abrupt aging surge in cortical and hippocampal neurons at middle-age (40-week) in mice. Failure in up-regulation of neuronal repair mechanisms to counter this aging surge in *Il33*^*−/−*^ mice may have led to chronic neuron death at old age. Therefore, neurodegeneration is initiated in *Il33*^*−/−*^ neurons probably just after the aging surge. There are two significances for our discovery of the aging surge. First, neurodegeneration in *Il33*^*−/−*^ mice is due to uncontrolled aging surge. It can be considered an accelerated aging process in neurons. This accelerated aging process causes slow and chronic neuron death, which is well reflected by a long period of time between the aging surge at 40 weeks and heavy neuron loss/behavioral changes after 60–80 weeks. In fact, chronic neurodegeneration with a long asymptomatic period followed by a stage with mild clinical symptoms is an important hallmark for human AD.^1^ Second, human late-onset AD is often diagnosed when massive neuronal death has already occurred, and effective therapeutic intervention is impossible.^1^ Therefore, identification of biomarker for early diagnosis is a medical priority. If a neuronal aging surge at middle age (45–50 years) exists in humans, it will be then a promising time point to search for biomarkers for early diagnosis of AD long before massive loss of neurons. Our model will be a useful tool in exploring these biomarkers.

From this study, we are able to propose a hypothesis for cause of late-onset AD. The aging surge at middle age causes damages to neurons. Stressed neurons may signal surrounding astrocytes, which, in turn, cleave nuclear IL33 to release cytokine IL33. With ST2 as receptor, IL33 upregulates DSB repairing and autophagic digestion to ensure ‘rejuvenation’ of the aged neurons. Thus, deficiency in IL33 or its associated signal pathway impairs neuronal rejuvenation, leading to accumulation of DSBs and incomplete autophagy, which are known to accelerate aging process in neurons. Some studies also showed that defective autophagy is responsible for accumulation of abnormal tau and amyloid.^50, 51^ As neurons are non-proliferative, rejuvenation of aged neurons is a prerequisite for a functional brain in elderly.^41, 42, 43^ Many studies have shown that repair of DNA damages and autophagic disposal of cellular wastes, for example, abnormal tau, are essential for neuronal rejuvenation.^6, 7, 8, 9, 10, 47^ Failed repair of stressed neurons leads to neurodegeneration in the cortex and hippocampus after middle-age and subsequent AD-like dementia at old age. To test our hypothesis in future, we need to address several questions. First, how does IL33 regulate repair mechanisms in aged neurons? Although it still remains unclear, our study suggests that cytokine IL33 and its receptor ST2 may be involved, because of presence of cytokine IL33 in normal brains (Figure 1d) and expression of ST2 mRNA in the cortex and hippocampus but not in other regions at middle age (unpublished data). Recent studies also revealed roles of other cytokines in AD.^14^ It will be interesting to examine cross-talk among these cytokines. Second, whether the aging surge and IL33 also play roles in AD development in transgenic mice with WT human genes? It is worthwhile to mention that transgenic mice with WT human APP or tau do not develop AD-like disease or amyloid plaque/tau deposition.^53, 54, 55^ It will be very interesting to test whether IL33 deficiency in those transgenic mice will cause AD-like disease as well as amyloid plaques/tau deposition. In conclusion, our study revealed a critical role of IL33 in repair of stressed neurons especially in the cortex and hippocampus after an abrupt aging surge. IL33 deficiency leads to uncontrolled neuronal aging, which in turn causes tau abnormality, neurodegeneration and AD-like disease at old age.
