# Interleukin33 deficiency causes tau abnormality and neurodegeneration with Alzheimer-like symptoms in aged mice

**DOI:** 10.1038/tp.2017.176

**Published:** 2017-08-01

**Authors:** C Carlock, J Wu, J Shim, I Moreno-Gonzalez, M R Pitcher, J Hicks, A Suzuki, J Iwata, J Quevado, Y Lou

**Correction to:** *Translational Psychiatry* (2017) **7**, e1164; doi:10.1038/tp.2017.142; published online 4 July 2017

The ninth author’s name was presented incorrectly. It should appear as J Quevedo.

