# Supplementary material for: Interleukin33 deficiency causes tau abnormality and neurodegeneration with Alzheimer-like symptoms in aged mice
Source: Transl Psychiatry. 2017 Jul 4;7(7):e1164–. doi: 10.1038/tp.2017.142 (PMC5538122; doi:10.1038/tp.2017.142)
Supplement: Supplementary Figures 1 and 2 [file tp2017142x1.docx]

**Supplementary Materials for**

**Interleukin33 deficiency causes Tau abnormality and neurodegeneration with Alzheimer-like symptoms in aged mice**

**
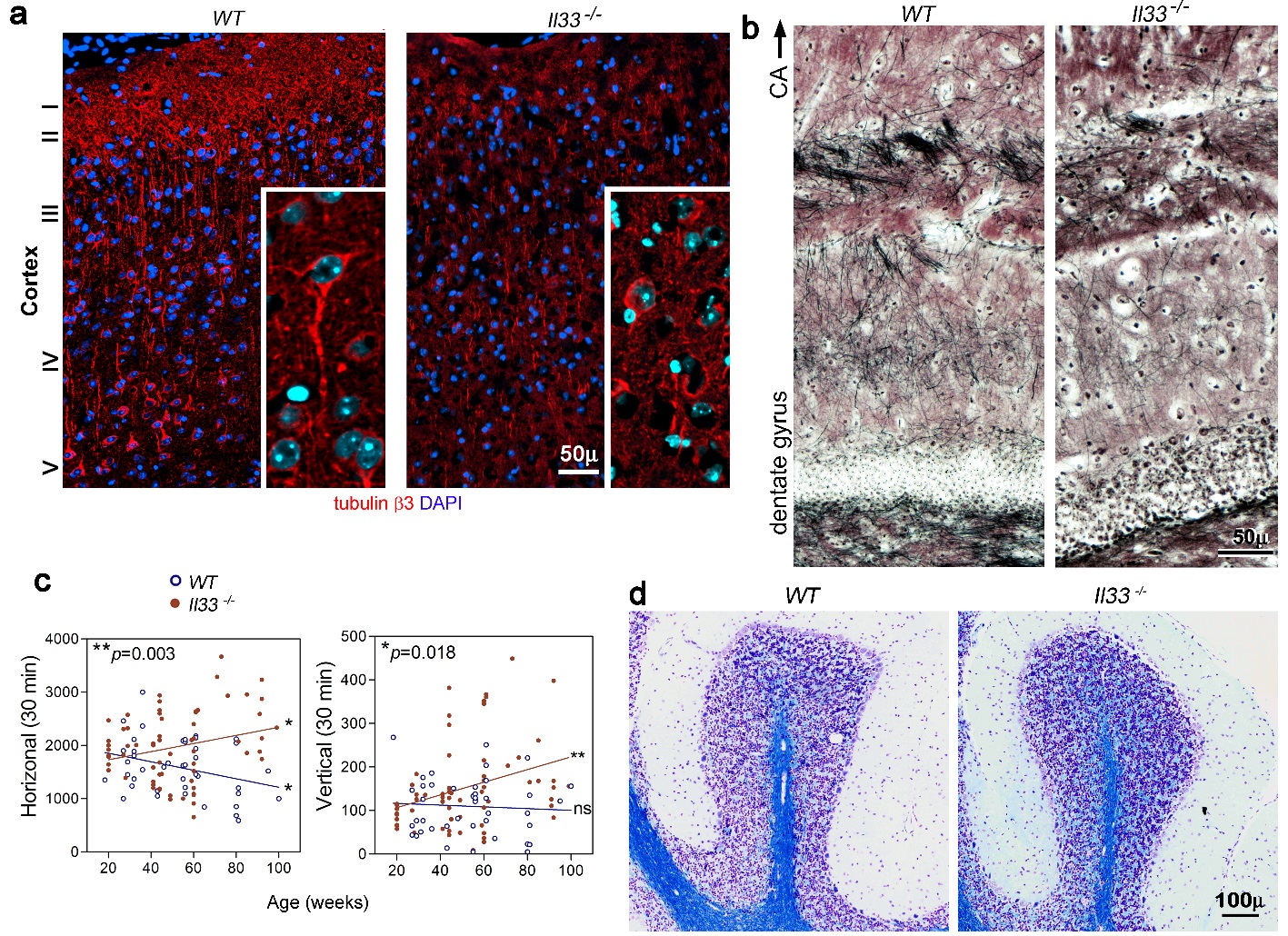
**

**Supplemental Figure 1.** *Il33^-/-^* mice show neurodegeneration in the cerebral cortex and hippocampus, and Alzheimer-like behavioral changes after 60 weeks. (**a**) Immunofluorescence reveals I to V layers in cerebral cortex of a WT mouse (left), but the layers are not recognizable due to loss of neurites in *Il33^-/-^* mice (right). Insets show loss of axons and dendrites in *Il33^-/-^* neurons. (**b**) Silver staining of hippocampus reveals loss of neurites in *Il33^-/-^* mice. The same regions from an age-matched WT are shown for comparison. (**c**) Regression analysis on locomotor activities from 20 to 90 week. *Il33^-/-^* mice show a significant increase in locomotor activities with age especially after 40-60 weeks. In contrast, WT mice show either no changes (in vertical), or a decrease (in horizontal) with age. (**d**) Crystal violet staining shows normal cerebella in aged *Il33^-/-^* mice (65 week) as compared to age-matched WT mice.

**
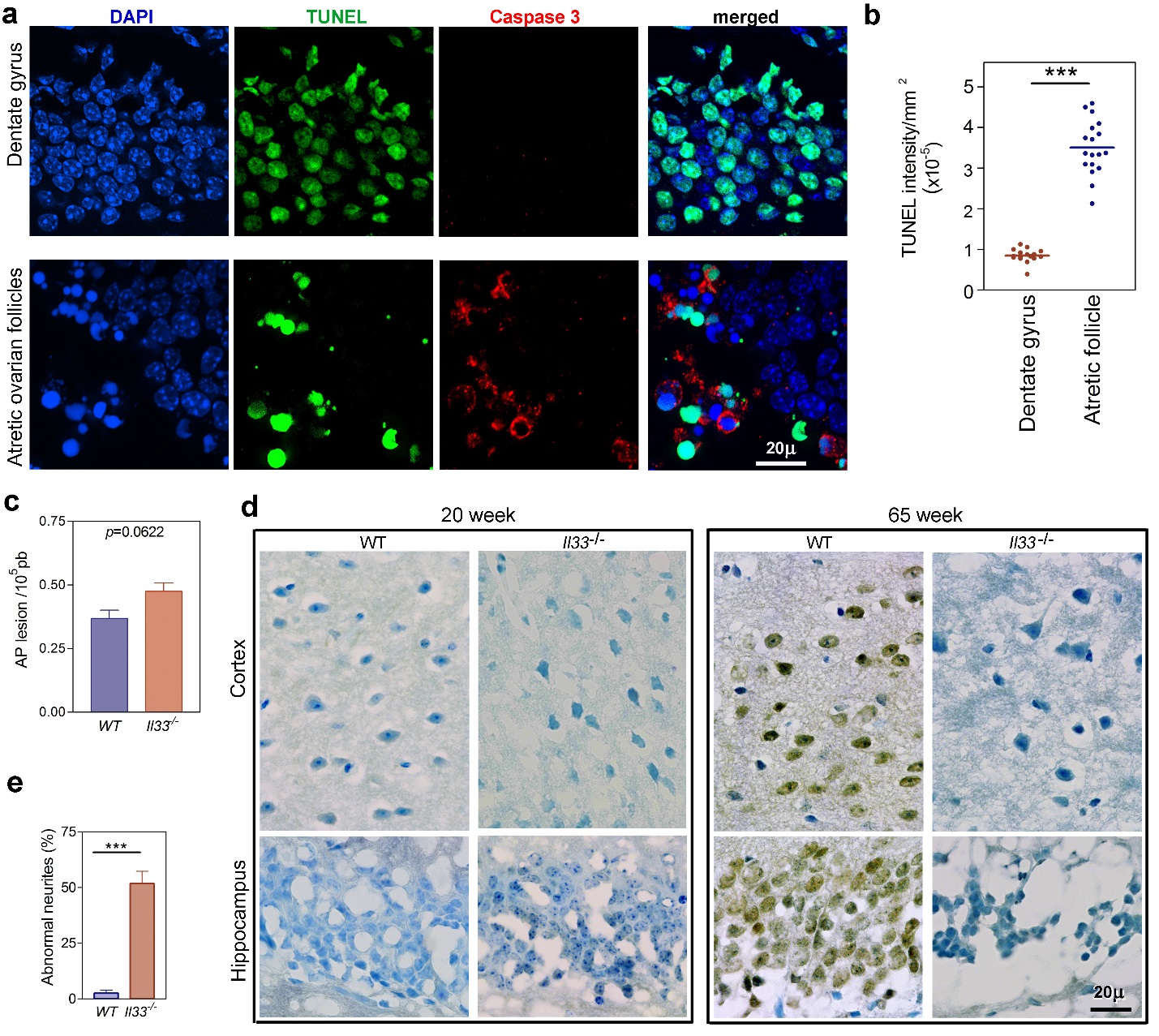
Supplemental Figure 2**. *Il33^-/-^* mice fail to repair DNA DSBs in cortical and hippocampal neurons. (**a**) Immunofluorescent comparison of TUNEL^+^ neurons in dentate gyrus of hippocampus of 40 week *Il33^-/-^* mouse with its own apoptotic ovarian cells reveals a much less intensity of TNUEL staining and absence of caspase3 in TUNEL^+^ neuron. Also note condensed chromosomal DNA (blue, DAPI staining) in apoptotic ovarian cells, but not in TUNEL^+^ neurons. (**b**) Statistical comparison of TUNEL intensity between TUNEL^+^ neurons and apoptotic ovarian cells shows a much lower intensity in TUNEL^+^ neurons. (**c**) Quantitation of AP sites caused by oxidative stress in cortical genomic DNA in *Il33^-/-^* and *WT* mice at 40 weeks shows a slightly elevated AP site in *Il33^-/-^* mice. However, the difference is not statistically significant. *n*=5. (**d**) Immunohistochemistry shows expression of PγH2AX in neurons in old WT mice (65 week), but not in young WT (20 week), nor all ages of *Il33^-/-^* mice. Counter-staining also reveals abnormal tissues, and loss of neurons in *Il33^-/-^* mice of 65-week old. (**e**) Percentages of neurites with vacuoles in WT and *Il33^-/-^* cortical neurons at 60 week based on five fields of electron microscopy.
